# The Impact of the Ebola Virus Disease Epidemic among Women in the Provinces of North Kivu and Ituri in the Democratic Republic of the Congo

**DOI:** 10.29245/2578-3009/2023/S3.1103

**Published:** 2023-05-12

**Authors:** Nkechi G. Onyeneho, Ngozi Idemili Aronu, Ijeoma Igwe, Joseph Okeibunor, Tieman Diarra, Julienne Ngoudougou Anoko, Mamoudou Harouna Djingarey, Zabulon Yoti, Dick Chamla, Abdou Salam Gueye

**Affiliations:** 1 University of Nigeria, Nsukka; 2 World Health Organization, Switzerland; 3 Independent Consultant, Mali; 4 Independent Public Health Expert, Niger

**Keywords:** Gender, Domestic division of labor, Epidemiology, Patients, Deaths, Men, Women, Ebola virus disease

## Abstract

Although an outbreak of the Ebola virus disease affects an entire population, women are more susceptible to the virus than men. Throughout the outbreaks of the Ebola virus disease in Central and West Africa, women have been impacted more significantly. Generally, over half of those who become ill are women. The situation is the same in terms of mortality. Further, the outcomes of the epidemic negatively affect women socially, as many become the heads of households following the loss of their spouses, which burdens them with new responsibilities. Women’s access to health services is also lowered, as the epidemic usually leads to fewer healthcare workers, impacting gynecological assistance. Consequently, women are more exposed to health problems, particularly during pregnancy. Several factors contribute to the greater exposure of women to the Ebola virus disease during an epidemic. First, female healthcare workers are at the frontline of the fight against the virus. Second, women’s duties in the domestic context increase their exposure to contamination, as they look after children and care for sick household members. Finally, women are responsible for several community duties such as public tasks and rituals. In the case of rituals, women undertake tasks such as undressing, washing, and dressing the deceased. Likewise, they engage in agricultural work and grocery shopping locally, as well as at cross-border markets. They also manage domestic chores such as fetching water in public places. Additionally, women have less access to information on the disease and its prevention and are thus more vulnerable. However, women’s vulnerability is less visible, since information on the epidemic and response is not gender specific. This is true for the number of suspected cases, confirmed cases, vaccinated people, alerts, contacts, contacts followed up, and screened travelers. It is therefore crucial to highlight the importance of gender in the response to the Ebola virus disease epidemic, as women are the primary victims.

## Introduction

Women account for most victims of the Ebola virus disease (EVD) epidemic in Central and West Africa. They have been more severely impacted than men in every outbreak of EVD in this region. Women get sicker than men and account for a larger number of deaths. In particular, women’s social and domestic responsibilities make them more vulnerable to infection^1^. They are the ones who care for sick household members and look after children, who are bathed, dressed, and fed by them. In most communities, women are also responsible for undressing, washing, and dressing the bodies of the deceased and for performing death rituals. This exposes them more extensively to the disease during an epidemic. Further, as women manage domestic chores, they come into greater contact with other people, for example, when fetching water from a public well or fountain and grocery shopping locally as well as at cross-border markets. Similarly, female healthcare workers are at the frontline of the fight against EVD. They meet potentially infected people and patients, which increases their exposure to contamination.

Data collected through interviews and questionnaires reveal the greater exposure of women than men to EVD^2,3,4^. However, information about the epidemic is not gender specific, particularly data on the number of suspected and cases, vaccinated people, alerts, contacts, contacts followed up, and screened travelers. Given the foregoing, it is crucial to highlight the importance of gender in the implementation of activities in response to the EVD epidemic and also to clearly understand gendered information about the response.

In this paper, we focus on women as the group most affected by the EVD epidemic. We then discuss the reasons for women’s greater exposure to the disease. Specifically, we explore and document the experiences and lessons around the response to the 10^th^ EVD outbreak in the North Kivu and Ituri provinces of the Democratic Republic of the Congo (DRC).

## Study Design and Methods

### Study design

In this study, we adopted a cross-sectional design with a mixed-method data collection technique. The cross-sectional design allowed multiple windows of data harvesting, while the mixed-method technique enabled us to use both quantitative and qualitative approaches. This guaranteed the integrity and robustness of the interpretations and conclusions drawn from this study.

#### Selection of the study area

The study was carried out in the North Kivu and Ituri provinces of the DRC, two of the country’s 26 provinces. Ituri is located northeast of the Ituri River and on the western side of Lake Albert. Its capital is Bunia, and it is home to the Ituri rainforest. Ituri has a high plateau (2000–5000 m) that has a large tropical forest but also savannah. The district has rare fauna, including the okapi, the national animal of the Congo. As for flora, an important species is mangongo, whose leaves are used by the Mbuti, a pygmy ethnic group, to build their homes. The population of the province is composed primarily of the Alur, Hema, Lendu, Ngiti, Bira, and Ndo-Okebo, with differing figures on which of these groups constitutes the largest percentage of the population. The Mbuti reside primarily in the Ituri rainforest near the Okapi Wildlife Reserve, although some have been forced into urban areas by deforestation, over-hunting, and violence. The Kilo-Moto gold mines are partly located in Ituri. At the beginning of the 21st century, petroleum reserves were found on the shores of Lake Albert by Heritage Oil and Tullow Oil.

North Kivu province borders Lake Kivu in eastern DRC, Ituri province in the north, Tshopo province in the northwest, Maniema province in the southwest, and South Kivu province in the south. To the east, it borders Uganda and Rwanda. The province consists of three cities—Goma (the capital), Butembo, and Beni—and six territories—Beni, Lubero, Masisi, Rutshuru, Nyiragongo, and Walikale. It is home to Virunga National Park, a World Heritage Site containing endangered mountain gorillas. Except for the heightened insecurity and isolation due to rebel activities, North Kivu shares similar demographics to Ituri. The province is politically unstable and has been one of the flashpoints of military conflicts in the region since 1998.

The 10^th^ EVD outbreak began on August 1, 2018, when it was confirmed that four cases had tested positive for Ebola virus in Kivu^5,6,7^. The outbreak later encompassed Ituri province, with the first case confirmed on August 13^8^. This outbreak started just days after the end of the 2018 Équateur province EVD outbreak^9,10^.

The affected province and general area are in the midst of a military conflict, which is hindering treatment and prevention efforts. The World Health Organization (WHO) described the combination of military conflict and civilian distress as a potential “perfect storm” that could lead to a rapid worsening of the outbreak^11^. Owing to the deteriorating situation in North Kivu and the surrounding areas, the WHO on September 27, 2019, raised the risk assessment at the national and regional level from “high” to “very high”^11^.

#### Study population

The study population comprised adults aged ≥18 years living in the community as well as response team members. A 2010 estimate put the population of North Kivu at 5,767,945. With an annual growth rate of 3.2%, the populations in 2019 were estimated to be 7,658,406 and 5,360,884 for the general and adult populations, respectively. A 2005 estimate put the population of Ituri at 4,037,561; hence, the 2019 populations were estimated to be 6,275,305 and 4,392,714 for the general and adult populations, respectively.

The response team consisted of over 10,000 people in different response pillars, including surveillance, risk communication, social anthropology, and vaccination. Others included infection prevention and control, treatment and care, and safe and dignified burial, as well as security, logistics, and administration.

#### Sample size estimation and sampling strategy

This was an exploratory study in which a sample of the study population was taken. With an assumed 50% chance of having accepting Ebola intervention at a confidence interval of 95%, and with an error margin of 5%, a sample size of 384 participants in each province was computed as necessary for the quantitative study. This was rounded up to 800 across both provinces to allow for losses. The final size of the sample depended on data saturation after an initial pair was collected from each category of respondents.

A multi-stage sampling technique was adopted to select the communities, households, and respondents in this study. Two administrative areas (the epicenters of the EVD outbreak in each province) were purposively selected. Ten communities were then randomly selected from each of these two administrative areas in the province.

The center of the selected community was the reference point where the team spun a pencil to determine the first route and first household. Thereafter, they moved to the right to pick the next household and then continued until the number of households to be sampled was reached. When there was a cul-de-sac, the steps were retraced, and a turn to the left and then one to the right were made, to continue the sampling process. Within a selected household, an adult (≥18 years) was randomly selected for inclusion in the study. The sex of the participants was carefully alternated; for example, if a man was selected in one household, the focus was on selecting a woman in the next household.

### Methods

The study was conducted using a mixed-method approach including qualitative and quantitative techniques. The data were gathered through in-depth interviews (IDIs), focus group discussions (FGDs), and surveys using structured questionnaires. Mixed-method studies require a strong focus on individuals rather than state actors^12^. Table 1 shows the distribution of the participants of the IDIs and FGDs by province.

For the FGDs, 8–12 people were selected for each session. A minimum of two FGDs were conducted in the selected communities, with separate sessions for men and women. Overall, eight FGDs were conducted in each province. A set of questions covering different thematic areas were developed to guide the discussions. The questions covered healthcare services in the community, EVD awareness and practices, and an assessment of the different pillars of the response interventions.

The IDIs were conducted in each community in which the FGDs were carried out. The IDIs were held with the community/opinion leaders in the selected communities and the team leaders of the response pillars. They were used to explore people’s opinions, views, attitudes, practices, and insights regarding the outbreak and response as well as the sociocultural factors that may influence their attitudes toward the response. The FGD guide was used for the IDIs to ensure focus on the thematic areas of interest.

A structured questionnaire was used to collect quantitative data from the households. The initial analysis of the IDIs and FGDs informed the final development of the questionnaire. It was categorized into five sections: sociodemographic data, perception of health problems in the community, knowledge of EVD, perceived epidemiology of EVD, and sources of information on EVD. It also included questions on communication and community engagement, infection prevention and control, vaccination, surveillance, treatment and care, safe and dignified burial, psychosocial issues, and logistics and security issues.

All the IDIs and FGDs were tape-recorded, and detailed notes were taken simultaneously, including verbal citations. The tape-recorded interviews were transcribed according to standard rules. Observations were also recorded to triangulate the quantitative data. The initial analysis of the IDIs and FGDs informed the final development of the structured questionnaire, as noted above, which further enhanced triangulation between the two datasets.

#### Training and pilot tests

All the instruments were translated into Swahili and French, the common languages spoken in the communities, and back-translated into English to ensure that all the meanings were clear. In each province, 10 research assistants with substantial experience in community interactive research and the use of qualitative and quantitative techniques as well as cultural sensibility were recruited. They were trained for three days in Beni and another three days in Bunia on the study objectives and use of the instrument for data collection. Training also included data entry into Atlas. ti (qualitative data) and EPI Info (quantitative data). The data analyst developed and pretested the template for data entry and analysis using the pilot test results.

In both provinces, a supervisor worked with the principal investigator to monitor data quality, advise on safety, and ensure that the research was conducted ethically, including the management of the informed consent procedures. The study was first conducted in Ituri and then in North Kivu. The lessons learned from Ituri were used to manage the process in North Kivu, which faces more security and logistics challenges than Ituri. Given the short study period, the data were collected using pencil and paper instead of a mobile device. The fieldwork took 20 days to complete in each province.

### Data management

All the quantitative data were double checked by the researchers before being entered into EPI Info and processed using SPSS. Descriptive statistics were used to determine and compare the proportions of the categories of respondents and indicators. Frequency tables and illustrations were used to present the data. While the quantitative results provided statistical conclusions, the qualitative results emphasized what was said and provided illustrative quotes that gave context and depth to the quantitative results.

### Ethical considerations

The principle of “do no harm” was adhered to in this study. Informed study approval was obtained from the province, local administration, community, and household, while informed consent was obtained from all the individuals that participated in the study. The WHO/AFRO Ethics Review Committee gave ethical approval for the study. All the researchers attended the mandatory training, which included a substantial discussion of ethical issues in research. Half the research assistants were women, ensuring that same-sex interviews could be conducted and the FGDs moderated. The assistants were also trained and mandated to comply with child protection and gender sensitivity standards during the data collection and visits.

## Results

### Women are the most affected by the epidemic

The IDIs showed that women are the most affected by EVD. This situation becomes clear in the words of the chief of the Boikene district of Beni:

In my opinion, women are the most exposed because they are the ones who handle the bodies of the deceased and frequently without knowing what caused that death. This attachment to others, or nostalgia, can bring them problems.

The chief of the pygmy camp in the same area agreed:

In my opinion, women can be the most contaminated by this disease because they are always with the children, perhaps the way children are around each other. When a child gets contaminated, the mother is the first to be exposed to the disease. The same is true for the healthcare of men in the hospital, which is done by the women.

The chief of Avenue Lubero in Butembo said that among the five people in his area who were affected, three were female (two women and one girl) and two were men. However, some people believe that it is mostly young people who are affected, irrespective of gender.

**Figure 1:**
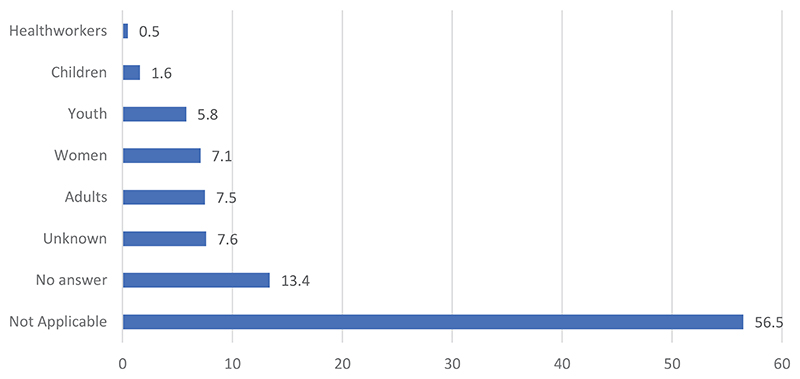
Categories of people perceived to be the most susceptible to EVD deaths: IDI data

**Figure 2:**
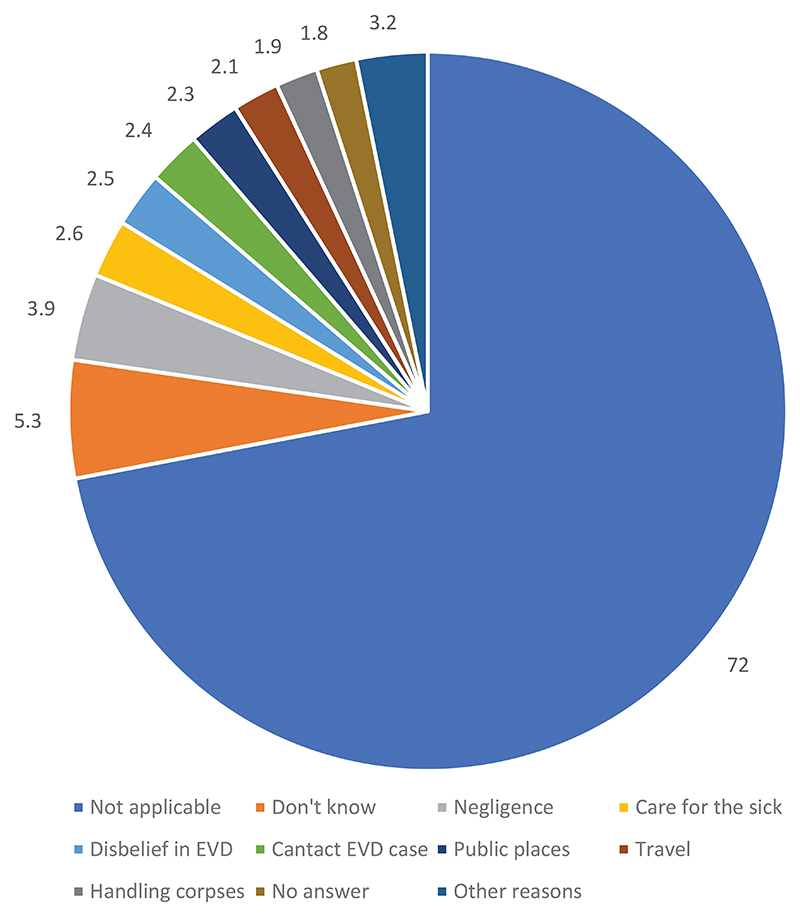
Perceived causes of death due to EVD: IDI data

Figure 1 reveals that those most affected by the disease are adults (7.5%), followed by women (7.1%) and youth (5.8%). The majority (56.5%) of the respondents held the view that there is no difference in the category of people most affected (not applicable). Moreover, 13.4% did not provide an answer, while 7.6% indicated that they could not tell which categories of people are the most affected. Figure 2 shows that 72.0% of the respondents could not respond to the question about their knowledge on EVD. More than 5% (5.3%) indicated that they did not know; 3.9% did not believe in EVD. People are exposed to the disease because of negligence, a lack of knowledge about the disease, disbelief in the existence of the disease, the decision not to follow hygiene measures, and the fact that they are frequenting public places, traveling, and handling the bodies of those who have died from EVD.

**Figure 3:**
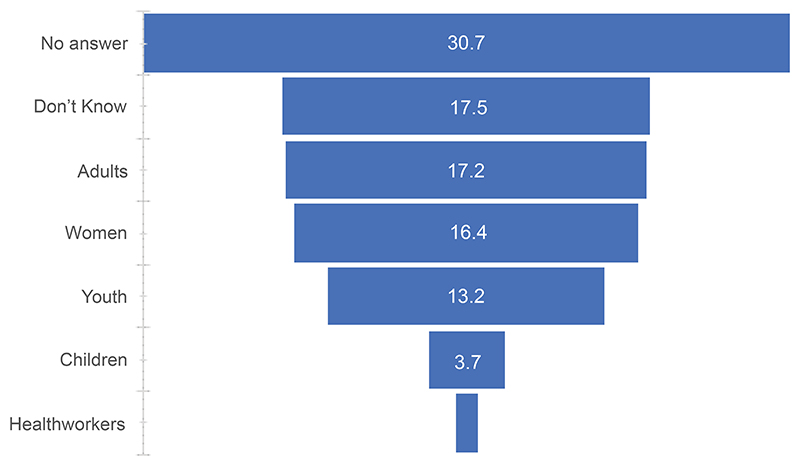
Categories of people perceived to be the most susceptible to EVD deaths: Survey data

**Figure 4:**
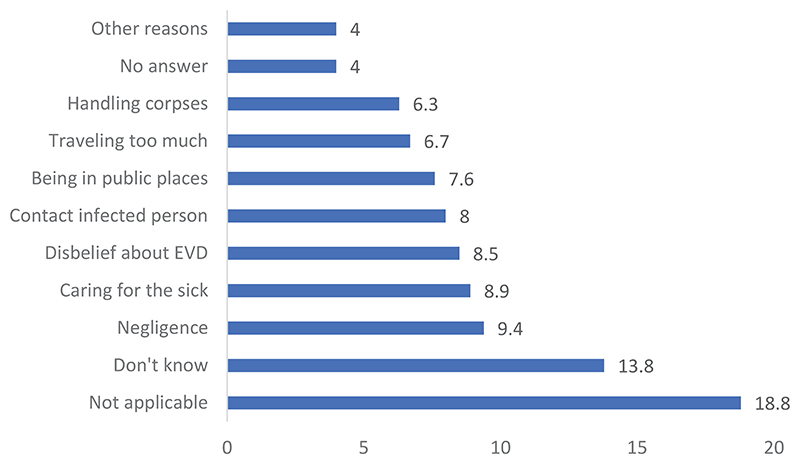
Perceived causes of death due to EVD: Survey data

Regarding the survey data, Figure 3 reveals that adults are perceived to be the most at risk of EVD deaths (17.2%), followed by women (16.4%), youth (13.2%), and children (3.7%). Health workers had the fewest answers (1.1%). Figure 4 reveals that the main causes of EVD deaths include negligence (9.4%) and caring for infected people (8.9%).

**Figure 5:**
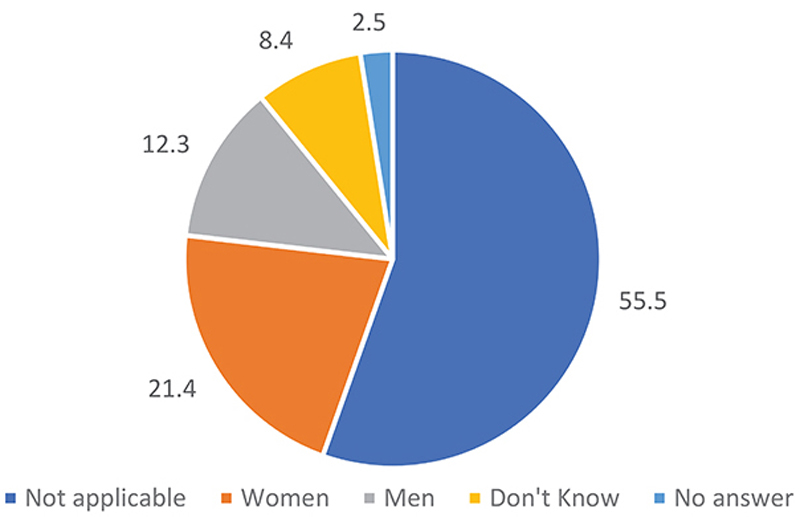
Most affected people by gender

Over a quarter of the interviewees believed that women are the most affected. If we do not account for unaffected communities, 48% thought that women are more affected, compared with 27.5% who thought that men are more affected (Figure 5). However, the village chiefs and neighborhood chiefs were unanimous in their agreement that women are the most affected (see the interview excerpts above).

### Reasons for women’s greater exposure to the disease

According to the chief of the Butama village in the health zone of Mandima, women are the most affected and account for the largest share of deaths from EVD. The chief stated,

It is women because they have more affection for others. Mothers take care of preparing the bodies of the deceased. They watch them until they are buried. Just like for the sick, women show more solidarity than men. They visit the ill, they wash their clothes, and sometimes they bathe them.

The chief of Avenue Masiki in Butembo agreed. According to him, women take care of the sick and are close to the dead during mourning. Thus, they can infect children or become infected when checking if a child has a fever. He stated,

The mother puts her cheek on the child’s cheek, touches it with her hand, or touches the child’s body. In case of death, the woman washes the corpse before burial. Imagine how many women became infected because they were unaware of the disease at the beginning of the epidemic.

The chief of the Bamako village also stated that women are the most exposed to EVD because, according to him, they carry the greatest burden of housework and are exposed to the illness through this work. He stated that when cooking, women might handle infected meat, and then transmit the disease to other family members. The chief of the Ngadi neighborhood spoke mostly of children (boys and girls) being the most affected. He mentioned four people affected in his neighborhood: three children and one man. By contrast, the chief of the Mabasele area felt that women are the most affected because of their activities outside the household, which increase the risk of meeting an infected person.

## Discussion and Conclusion

The results of this study revealed that women are the most at risk of EVD, as previous studies have highlighted^13^. Kapur (2020) found that over half of the people who have died from EVD in the DRC are women and that children are also more affected than men. Other studies across Africa have similarly found that more women are infected with EVD than men because of their daily activities^14,15^. Further, mortality rates are even higher among pregnant women^15^.

In the first Ebola epidemic in 1976 in the DRC, women were more affected than men^11^. This was also the case during the 1995 epidemic in the DRC^16^, as well as in Liberia, Guinea, Sierra Leone^17^, and Nigeria, with women accounting for 55% of cases^3^. In Uganda, during the epidemics of 2000 and 2001, women again accounted for the highest number of victims of the disease^18,14^. Across most of Africa, women take care of sick household members. During the seventh Ebola outbreak in the DRC, the index case was a pregnant woman. Of the 69 patients, 36 were women and 33 were men, and 74% of the deceased were women^16^.

In addition to being more affected by the disease, women are more marginalized and less involved in Ebola response activities. Consequently, surveillance, contact monitoring, and social mobilization teams consist of only few women. Further, some communities restrict women’s participation in public roles^19^. Women are also more stigmatized and become the scapegoats of traditional social structures^19^.

Nevertheless, in this study, some of the respondents considered that healthcare workers to be the main victims of EVD. Several reasons were mentioned (see Figure 4). Negligence was a reason for exposure. However, some stated that women are more affected because they are more frequently the key caregivers of people who fall ill or have more contact with them. This concurs with the opinions provided by the community leaders during the IDIs. Other studies have also found that women are often in public places, travel often, and handle the bodies of the deceased^18^.

The epidemic can also affect women’s health, as attendance at centers for reproductive healthcare services was found to have dropped by 30% in some countries after an outbreak^3^. One study in Guinea found that before the outbreak of the epidemic, maternal health had improved in terms of assisted deliveries and antenatal visits, but that these trends were reversed during the epidemic^20^. Likewise, after the outbreak, the vaccination rate of children decreased^20^. The same study found a significant decline in maternal and child health indicators. During the ninth epidemic in the DRC, the fear of contracting the virus was also at the root of the drop in attendance at health centers. Furthermore, the epidemic had a social impact on women, who also lost confidence in the health system^21^. In some cases, such as in Sierra Leone, women became heads of their households following the loss of their spouses^17^.

Women have less access to information on EVD than men, which increases their risk of contracting the disease^22^. Women are generally the primary caregivers for the sick in their households (e.g., mothers often travel with their sick children to seek treatment), and their occupations also make them more susceptible and exposed to the disease^13^. Women’s work also exposes them to the disease, as they must cross borders in search of a livelihood^3^. Women are also involved in rites such as burials (e.g., washing the body of the deceased^13^), which entail being physically close to the deceased^23^, including those infected with EVD. In Uganda, women undress, wash, and dress the bodies for burial^14^. Social norms such as shaving the corpse may also expose women to the disease^24^.

In some cases, the domestic division of labor, together with sociocultural determinants, result in women and girls being further exposed^1^. Some have even spoken about the social and economic vulnerability of women, as they must travel around to complete their daily activities, such as fetching water and going to the market^1^. In many societies, they also share their meals and wash the clothes of other household members, including washing and dressing children.

In conclusion, the IDIs and questionnaire survey carried out in this study show that women are the most affected by the EVD epidemic. They become more ill than men, and a larger number of them die from the disease. However, epidemiological data do not account for the gender-related difference in the number of suspected and confirmed cases, vaccinated people, alerts, contacts, contacts followed up, and screened travelers. This study thus bridges a gap in the literature, as there is an urgent need for gender issues to be placed in the mainstream of discussions and debates about epidemics.

## Figures and Tables

**Table 1:** Distribution of the participants of the in-depth interviews (IDIs) and focus group discussions (FGDs) by province.

Target	North Kivu				Ituri			
	Butembo		Beni		Mbuti		Bunia	
	IDI	FGD	IDI	FGD	IDI	FGD	IDI	FGD
Pillar leads	All		All		All		All	
Pillar members	2/pillar		2/pillar		2/pillar		2/pillar	
Community leaders^1^	≥2/community		≥2/community		≥2/community		≥2/community	
Leader of survivor groups	≥2/community		≥2/community		≥2/community		≥2/community	
Male adults		≥2 groups		≥2 groups		≥2 groups		≥2 groups
Female adults		≥2 groups		≥2 groups		≥2 groups		≥2 groups
Male youth		≥2 groups		≥2 groups		≥2 groups		≥2 groups
Female youth		≥2 groups		≥2 groups		≥2 groups		≥2 groups
Survivors		≥2 groups		≥2 groups		≥2 groups		≥2 groups

The community leaders in Table 1 include religious, traditional, political, and opinion leaders.

## Data Availability

The data that support the findings of this study are not publicly available because they contain information that could compromise the privacy of the research participants. The data are available from the corresponding author (Joseph Okeibunor) upon reasonable request.
